# Characteristics, clinical laboratory, histopathology, and outcomes of glycogenic hepatopathy in children

**DOI:** 10.1002/jpr3.12046

**Published:** 2024-02-05

**Authors:** Chaowapong Jarasvaraparn, Iván A. González, Kyla M. Tolliver, Nadine G. Haddad, Jean P. Molleston

**Affiliations:** ^1^ Division of Pediatric Gastroenterology, Hepatology and Nutrition Indiana University School of Medicine/Riley Hospital for Children Indianapolis Indiana USA; ^2^ Riley Hospital for Children at IU Health Indiana University School of Medicine Indianapolis Indiana USA; ^3^ Department of Pathology and Laboratory Medicine Indiana University School of Medicine Indianapolis Indiana USA; ^4^ Division of Pediatric Endocrinology and Diabetes Indiana University School of Medicine/Riley Hospital for Children Indianapolis Indiana USA

**Keywords:** diabetes mellitus, liver biopsy, poor glycemic control

## Abstract

**Introduction:**

Glycogenic hepatopathy (GH) is a rare complication of type I diabetes mellitus (DM1), resulting in abnormal deposition of glycogen in the liver due to poor glycemic control. Clinical characteristics and natural history of GH are not completely understood in children. In this study, we investigated clinical, biochemical, histologic parameters and outcomes in children with GH.

**Method:**

This was a retrospective review of patients less than 18 years old diagnosed with GH and DM. GH was confirmed on liver biopsy. Medical records were reviewed for clinical presentation, laboratory tests, and clinical outcomes. Liver biopsy findings were reviewed by a pediatric pathologist (I. A. G.).

**Results:**

Nine children were diagnosed with GH and type 1 DM. The median age at diagnosis of GH was 16 (IQR 14.5−17) years. Duration of diagnosis of DM until GH diagnosis was 7 (IQR 5−11) years. The median frequency of diabetic ketoacidosis before GH diagnosis was three times (IQR 2−5.25). Peak Aspartate transaminase (AST) and Alanine transaminase (ALT) ranged from 115 to 797, and 83−389 units/L, respectively. Only two children had mild fibrosis. Seven of nine had steatosis without steatohepatitis. There was no correlation between glycosylated hemoglobin (HbA1c), or other laboratory tests and liver fibrosis on biopsy. HbA1c was 11.2 (IQR 10.2−12.8) at GH diagnosis and 9.8 (IQR 9.5−10.8) with normalization of liver enzymes.

**Conclusion:**

GH appears to be related to poor glycemic control in teenagers with long‐term diabetes. GH presents with high to very high aminotransferase especially AST > ALT and resolves with modestly improved glycemic control. Diffuse hepatocyte swelling, steatosis, minimal fibrosis without hepatocyte ballooning or lobular inflammation are most common histological features.

## Abstract (highlights)

**What is Known:**

- Glycogenic hepatopathy (GH) is a rare complication of poorly controlled diabetes mellitus (DM) characterized by elevated aminotransferases and hepatomegaly.
- Elevated aminotransferases improve drastically after glycemic control in diabetes.

**What is New:**

- GH occurs after years of diabetes (7, IQR 5−11 years) and recurrent diabetic ketoacidosis in teenagers.
- Liver biopsy in GH can show hepatocyte swelling with minimal liver fibrosis and steatosis without steatohepatitis.
- GH tends to have aspartate transaminase‐predominant elevation of aminotransferase.
- GH resolves with only very mild improvement in glycosylated hemoglobin, indicating continued poor glycemic control.

**Abbreviations**
ALT: alanine transaminase
APRI: aspartate aminotransferase‐to‐platelet ratio index
AST: aspartate transaminase
DM: diabetes mellitus
FIB‐4: Fibrosis‐4
GH: glycogenic hepatopathy
HbA1c: glycosylated hemoglobin

## 1 INTRODUCTION

Glycogenic hepatopathy (GH) is a rare complication of poorly controlled diabetes mellitus (DM) seen in children and young adults. This is characterized by a reversible accumulation of excess glycogen in hepatocytes, causing transient or reversible liver injury manifested by elevated liver aminotransferases and hepatomegaly. Excessive glucose in sinusoids is rapidly taken up by hepatocytes, followed by conversion of glucose to glycogen, which is trapped within the liver. Glycogen metabolism is controlled by glycogen phosphorylase after feeding, so elevated glucose and insulin levels in poorly controlled DM decrease glycogen phosphorylase activity in hepatocytes, leading to the accumulation of glycogen in hepatocytes.^1^, ^2^ Mauriac syndrome was first described in 1930 in children presenting with GH, growth retardation, hepatomegaly, cushingoid features, and delayed puberty.^1^, ^2^ GH is mostly found in patients with longstanding poorly controlled type 1 DM and rarely in type 2 DM^1^, ^2^ in both children and adults. GH can only be diagnosed by liver biopsy on routine histology using hematoxylin and eosin stain which shows a diffuse pale staining of the hepatocytes corresponding to the glycogen accumulation which can be highlighted with a Periodic acid‐Schiff (PAS) stain and is digested with a PAS with diastase (PASD) stain.^1^, ^2^, ^3^ The condition is rare and can lead to extensive evaluation due to the degree of aminotransferase elevation.

There is a scarcity of studies, case reports, and case series^4^, ^5^, ^6^, ^7^, ^8^ in children with GH, so the pathophysiology of GH has not been well characterized in children. The aim of this study was to describe the clinical, biochemical, and histologic profile of children with GH.

## 2 METHODS

This was a retrospective case series at Riley Hospital for Children/Indiana University School of Medicine. Cases were patients less than 18 years old diagnosed with GH and DM. Children with GH were identified from liver biopsy. Patients with known chronic liver disease or patients in whom elevated liver enzyme could be attributed to other causes were excluded. Cases were identified by searching the term “glycogenic hepatopathy” and a combination of “glycogen” and “liver” in the pathology database, and the terms “elevated liver enzyme,” “hepatomegaly,” and “diabetes mellitus” from the electronic medical records database from ICD 9 and 10 codes with dates ranging from January 2010 to April 2023. All patients had negative evaluations for chronic liver disease such as viral infection, autoimmune hepatitis, celiac disease, Wilson disease, metabolic liver disease, hemochromatosis, and so forth. The study was approved by the Indiana University Institutional Review Board.

### 2.1 Clinical data

Demographic and clinical variables included age at presentation of DM and GH, gender, height/weight/BMI z score, imaging (ultrasound or computerized tomography scan), and usage of multiple doses of insulin or insulin pump. Clinical variables comprised duration of DM, time of finding of elevated liver enzyme, and hepatomegaly on physical examination and/or abdominal imaging. Laboratory tests including glycosylated hemoglobin (HbA1C), aspartate transaminase (AST), alanine aminotransferase (ALT), gamma‐glutamyl transferase (GGT), platelet, total bilirubin, albumin, prothrombin time (PT), and international normalized ratio (INR) were reviewed at the time of liver biopsy or as close as possible to the time of liver biopsy (3 months before or after liver biopsy). We used noninvasive biomarkers of liver fibrosis including AST‐to‐platelet ratio index (APRI), which was calculated using a reference upper limit AST of 40 U/L,^9^ and Fibrosis‐4 (FIB‐4), which was calculated using published formulas.^10^ APRI = (AST in IU/L)/(AST Upper Limit of Normal in IU/L)/(Platelets in 10^9^/L) and FIB‐4 Score = (Age x AST)/(Platelets in 10^9^/L x √(ALT)).

Liver biopsy findings including presence of inflammation, megamitochondria, steatosis, hepatocyte swelling, and fibrosis were reviewed by a pediatric pathologist (I. A. G.). Liver biopsies were processed by our institutional protocol in which for all biopsies 2 H&E stain levels are performed as well as the following special stains Masson trichrome, reticulin, iron and PASD. All stains were performed with appropriate controls. The degree of fibrosis was evaluated using the modified Scheuer system^11^ in which a semiquantitative assessment is used consisting of no fibrosis, fibrous portal expansion, periportal fibrosis, septal fibrosis, and cirrhosis. In addition, the presence or absence of pericellular fibrosis was noted. Steatosis was evaluated and when present its grade and the present of steatohepatitis was determined according to stablished criterion.^12^ Hepatocyte swelling was assessed semiquantitative as mild (<33%), moderate (33−66%) and severe (>66%).^3^

### 2.2 Outcomes

Follow‐up end points were chosen depending on physicians' decisions and were classified as “normalized liver enzyme” or “normalized hepatomegaly” and duration of normalized liver enzyme or normalized hepatomegaly since diagnosis of GH were recorded. Outcomes including cirrhosis, portal hypertension, and liver‐related events or death were assessed.

### 2.3 Statistical analysis

Results are presented as median and 20% and 75% interquartile for continuous variables, or frequencies (percentage) for categorical variables. Student *t*‐test was used to compare the continuous variables. Pearman correlation coefficient was used to evaluate the association between two continuous variables. P‐value less than 0.05 was used to determine the significance of results. SPSS was used for all statistical analyses (version 28, SPSS Inc.).

## 3 RESULTS

Nine children were diagnosed with GH and type 1 DM. The median age at diagnosis of GH was 16 (IQR 14.5−17) years. They were mostly male (5/9; 55.56%), and all were Caucasian. All children presented with elevated aminotransferases and hepatomegaly on imaging. Four children had elevated aminotransferase during diabetic ketoacidosis admission, which persisted after discharge from hospital; three children were found by their primary care providers or at an emergency visit to have elevated aminotransferase during evaluation for abdominal pain; and for two children, it was unclear why their aminotransferase levels were checked or the reason they had imaging performed. Median age at diagnosis of type 1 DM was 9 (IQR 5−10.5) years. Duration of diagnosis of DM until GH diagnosis was 7 (IQR 5−11) years. All subjects were on standard multiple dose injections. No child was on an insulin pump at diagnosis of GH. The median HbA1c, AST, ALT, total bilirubin, INR and platelet count at diagnosis of GH were 11.2 (IQR 10.1−12.8), 311 (IQR 115−797), 199 (IQR 83−389), 0.6 (IQR 0.4−1.05), 0.92 (IQR 0.85−0.96), 334 (IQR 275−392) x 10^3^/μL, respectively. All children were diagnosed with diabetic ketoacidosis before diagnosis of GH. The median frequency of diabetic ketoacidosis before GH diagnosis was 3 times (IQR 2−5.25).

Children were shorter compared to reference data. The median z score of height was −0.3 (IQR −1.9 to 0.27). Two children (22.2%) were diagnosed with growth failure (z score height < −2). No child had delayed puberty, cushingoid features, or obesity at GH diagnosis. Laboratory tests were reported in Table 1. No child had synthetic liver dysfunction or thrombocytopenia (platelet counts < 150,000 μL). All had normal albumin and total bilirubin level. Eight children had AST/ALT ratio > 1. There was no correlation between AST, ALT, or GGT and HbA1c. Two children had splenomegaly from ultrasound at GH diagnosis which had resolved at follow up visit.

**Table 1:** Clinical, biochemical, and histological characteristics and outcomes of children with glycogenic hepatopathy (GH).

Characteristics	Values
Age at GH diagnosis (years, median, IQR)	16 (14.5−17)
Age at DM type 1 diagnosis (years, median, IQR)	9 (5−10.5)
*Gender (%)*	
‐ Male	5 (55.56)
‐ Female	4 (44.44)
*Race (%)*	
‐ Caucasian	9 (100)
Weight z‐score (median, IQR)	0.5 (−0.75 to 1.14)
Height z‐score (median, IQR)	−0.3 (−1.9 to 0.27)
Body mass index z‐score (median, IQR)	0.83 (0.24−1.0)
Growth failure (%)	2/9 (22.2)
*Laboratory tests at GH diagnosis*	
HbA1c (%, median, IQR)	11.2 (10.1−12.8)
AST (units/L, median, IQR)	311 (115−797)
ALT (units/L, median, IQR)	199 (83−389)
AST/ALT ratio	1.51 (1.25−1.61)
GGT (units/L, median, IQR)	67 (50−180)
Albumin (units/L, median, IQR)	3.8 (3.45−4.3)
Total bilirubin (units/L, median, IQR)	0.6 (0.4−1.05)
Prothrombin time (seconds, IQR)	10 (9.3−10.55)
International Normalized Ratio (IQR)	0.92 (0.85−0.96)
Platelet (x10^3^/uL, IQR)	334 (275−392)
Multiple dose injection of insulin (%)	9/9 (100)
Duration of diabetes mellitus (years, median, IQR)	7 (5, 11)
*Noninvasive fibrosis score*	
APRI	2.4 (0.9−6.35)
FIB‐4	0.83 (0.56−1.92)
*Imaging (%)*	
Ultrasound	6/9 (66.7)
Computerized tomography scan	2/9 (22.2)
No imaging	1/9 (11.1)
Hepatomegaly	8/8 (100)
Splenomegaly	2/8 (25)
*Histological characteristics (%)*	
Fibrosis scores	
‐ 0 (no fibrosis)	7 (77.8)
‐ 1 (perisinusoidal fibrosis)	1 (11.1)
‐ 2 (periportal fibrosis)	1 (11.1)
*Steatosis scores*	
‐ 0 (nonsignificant steatosis)	2 (22.2)
‐ 1 (mild steatosis)	7 (77.8%)
Follow up	7/9 (77.7%)
Duration of follow up (years, median, IQR)	6 (3.12−12.25)
Normalized aminotransferases at follow up visit (%)	5/7 (71.4)
Aminotransferases were not available at follow up visit (%)	2/7 (28.6)
Duration to resolution of elevated aminotransferase (years, median, IQR)	2 (1.25−4.5)
Resolution of hepatomegaly (%)	4/7 (57.1)
No data resolution of hepatomegaly (%)	3/7 (42.9)
Duration to resolution of hepatomegaly (years, median, IQR)	5 (1.2−7)
Normalized aminotransferases and resolution of hepatomegaly (%)	4/7 (57.1)
HbA1c at normalized liver function test (%, median, IQR)	9.8 (9.5−10.8)

Abbreviations: ALT, Alanine transaminase; APRI, aspartate aminotransferase‐to‐platelet ratio index; AST, Aspartate transaminase; FIB‐4, Fibrosis‐4; GGT, Gamma‐glutamyl transferase.

**Figure 1:**
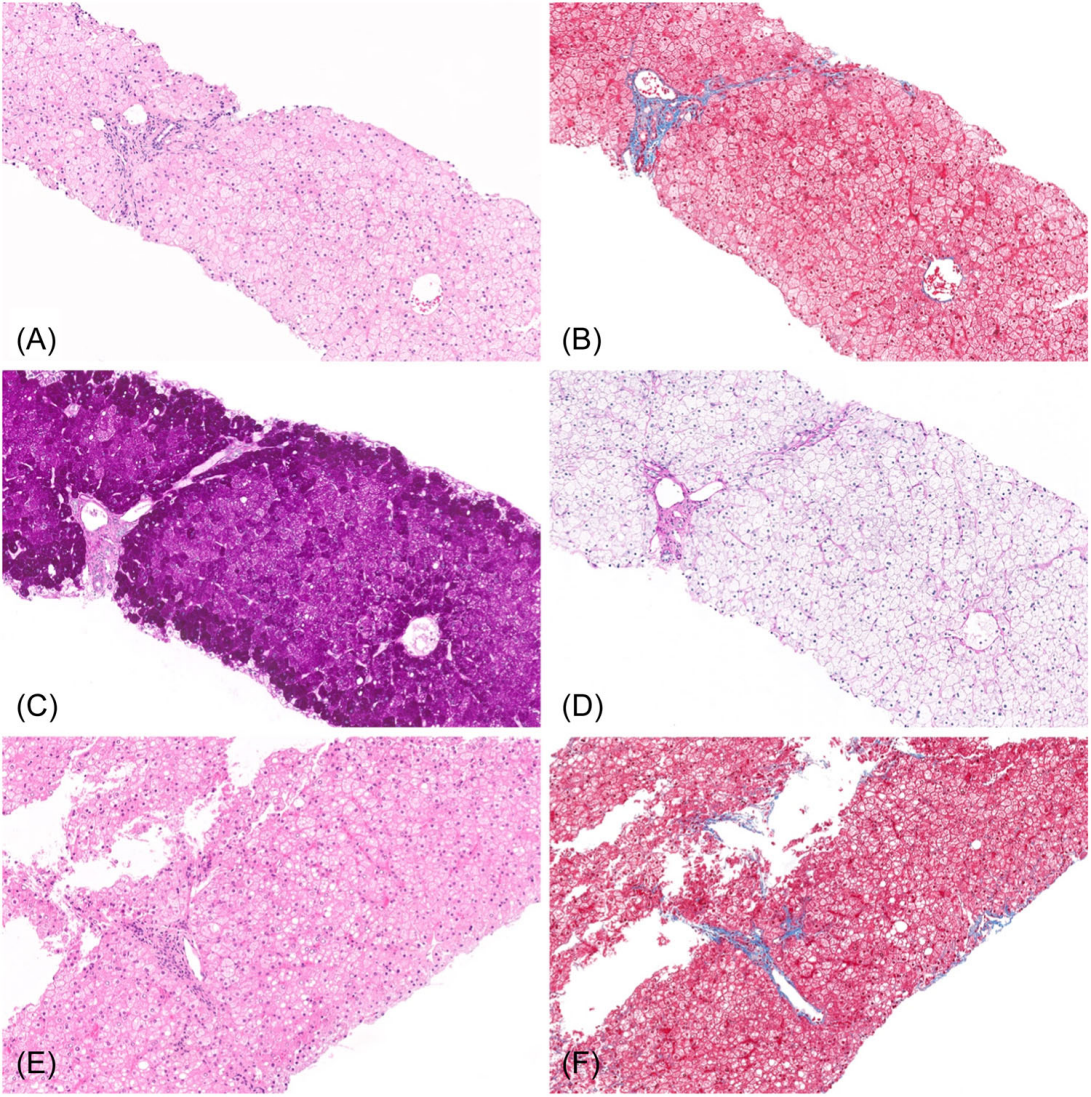
Liver biopsy of patients with glycogenic hepatopathy. Biopsy from a 14‐year‐old girl showing diffuse glycogen accumulation within hepatocytes without associated portal or lobular inflammation (A, H&E stain) and without increase in fibrosis (B, Trichrome stain). The hepatocellular glycogen is highlighted with a PAS stain (C) and is diastase sensitive (D, PASD stain). Separate biopsy from a 17‐year‐old boy with diffuse glycogen accumulation within hepatocytes as well as mild macrovesicular steatosis (E, H&E stain) with focal and minimal perisinusoidal fibrosis (F, Trichrome stain). PASD, periodic acid‐Schiff with diastase.

All children underwent liver biopsy within 6 months of finding elevated liver enzymes, and for seven slides were available for review by study pathologist. For the other two cases the pathologic information was extracted from the pathology reports which were rendered by a liver pathologist during routine clinical care. All liver biopsies showed an intact liver architecture with most of the cases showing no increase in fibrosis (77.8%), periportal fibrosis in one case and another focal perisinusoidal fibrosis in another. All cases showed a diffuse hepatocyte swelling characterized by a pale staining of their cytoplasm with cytoplasmic rarefication and accentuation of the cell membranes, and glycogenated nuclei (Figure 1). In seven cases (77.8%) mild macrovesicular steatosis was seen without features of steatohepatitis and none of the cases showed microvesicular steatosis, hepatocyte ballooning, or lobular inflammation. There was no correlation between AST/ALT ratio, APRI or FIB‐4, and liver fibrosis from biopsy.

After GH diagnosis, there were two children lost to follow up and two others for whom liver aminotransferase levels were not available at follow up visits. Median duration of follow up was 6 (IQR 3.12−12.25) years. At follow up, 5 children (5/7; 71.4%) had normalized aminotransferases and median time to resolution of elevated aminotransferase was 2 (IQR 1.25−4.5) years. Four children (4/7; 57.1%) had resolution of hepatomegaly on follow up imaging and median duration to resolution of hepatomegaly was 5 (IQR 1.2−7) years. Median HbA1C at time of normalized transaminases was 9.8 (IQR 9.5−10.8), which was lower than HbA1C at GH diagnosis (11.2, IQR 10.1−12.8) but not significantly (*p* = 0.14). Median HbA1c at the recent follow up was 11.73%. No child had liver cirrhosis, portal hypertension, or liver‐related events such as gastrointestinal bleeding, acute liver failure, hepatic encephalopathy, or death during follow up visits. For the median follow up at 6 years after diagnosis, seven children did not have any recurrent elevated liver enzyme or hepatomegaly and two were lost to follow up.

## 4 DISCUSSION

DM is associated with various structural and functional liver abnormalities such as elevated liver enzyme and hepatomegaly. Poorly controlled insulin‐dependent Type 1 DM with GH has been recognized as a cause of elevated liver enzyme and hepatomegaly since first reported in 1930.^13^ Based on previous reviews, 98% of GH was found in DM type 1, while 2% was caused by DM type 2.^1^ True incidence and prevalence of GH are unknown, especially in children. Misdiagnosis with other liver complications of type 1 DM such as nonalcoholic fatty liver disease has been described.^14^ It is not clear why some children with poorly controlled diabetes develop GH and others do not. Currently, liver biopsy is the gold standard to diagnose GH.

The pathogenesis of GH is excess glycogen accumulation in the liver in poorly controlled DM.^1^ The liver takes up glucose after feeding and stores as glycogen, maintaining the balance between glycogenosis and glycogenolysis.^1^ Subsequent hyperglycemia in poorly controlled DM enhances conversion of glucose to glycogen by glycogen synthase. Our case series confirmed association of poorly controlled DM with HbA1c at 11.2% but showed clinical resolution of GH with persistent HbA1c over 9%. Moreover, Mukewar et al. reported a higher frequency of diabetic ketoacidosis episodes in patients with GH compared with DM type 1 without liver disease.^15^ Persistent hyperglycemia and excess insulin administration during diabetic ketoacidosis treatment in type 1 DM are hypothesized to be metabolic preconditions for glycogen deposition in the liver which can damage hepatocytes.^14^

The description of Mauriac syndrome includes growth failure, delayed puberty, hepatomegaly, and cushingoid features in children with poorly controlled type 1 DM.^4^ We reported only two children with growth failure but no features of delayed puberty or cushingoid features. The remaining children in this study were neither significantly overweight nor malnourished. We do not believe that GH in poorly controlled DM1 is synonymous with Mauriac syndrome. Current better long‐acting insulins and improved glycemic control glycemia may contribute to fewer patients with typical features of Mauriac syndrome presently.

GH tended to have AST‐predominant aminotransferase elevation. AST/ALT ratio > 1 is independently associated with severe fibrosis (fibrosis stage 3 and cirrhosis) in adults with hepatitis C, nonalcoholic hepatitis, and alcoholic hepatitis.^16^, ^17^, ^18^ Eight of nine children had AST/ALT ratio > 1 in this study. This could be related to transient ischemia from compression of the sinusoids by glycogen obstructing blood flow and resolultion after controlling DM.^19^ There was no correlation between AST/ALT ratio, APRI or FIB‐4, and liver fibrosis from biopsy in this study.

It is notable that liver biopsies in this series had only mild liver fibrosis (fibrosis stage 1 or 2). Moreover, APRI and FIB‐4 are widely validated noninvasive scores for detecting liver fibrosis or cirrhosis of other liver etiologies.^9^, ^10^ However, APRI and FIB‐4 are not good noninvasive liver fibrosis scores of GH in this study. Torbenson et al. reported mild steatosis (2/14; 7%) in children and adults with GH ^3^ which was in contrast to this study showing 7/9 (77.8%) with steatosis from liver biopsy in lean children without steatohepatitis features. This steatosis finding confirmed the metabolic preconditions of poorly controlled hyperglycemia for glycogen deposition in the liver. Moreover, there was some fibrosis in this study, which could develop into more advanced liver fibrosis in the future, but we do not repeat liver biopsies in the current clinical practice.

One study in children found 54.8% had normalized aminotransferases and reduction to normal liver size at follow‐up ^4^ which was similar to this result of 57% (four from seven children with follow up visits). Aminotransferases followed the trend of the children's HbA1c in our study, which was similar to the previous studies.^4^, ^15^ Hepatomegaly can sometimes develop within days to weeks and can improve rapidly once the DM is under control,^20^ a finding which this series confirms. Two children had normalized splenomegaly at follow up. We speculated that this could be transient sinusoidal portal hypertension caused by glycogen obstruction. There was one case report of GH with ascites in a child, and ascites improved significantly with adequate control of DM. The pathogenesis of transient ascites in that case was unknown.^21^ To date, GH does not lead to adverse long‐term outcomes such as cirrhosis or portal hypertension.^14^ Advanced or chronic liver disease or complications were not seen in the present series.

Limitations of this study were the retrospective design lack of follow up on two children. Information on laboratory tests and imaging was not available for all patients. Some children did not repeat aminotransferases or liver imaging after diagnosis of GH. Second, two children did not have liver biopsy slides for review, requiring extraction of histologic features from the liver biopsy report. All patients obtained liver biopsies at an early stage of GH after finding elevated liver enzyme or hepatomegaly. There were no follow up liver biopsies, so the liver biopsies might be from an early stage of GH disease, which might not be accurate to evaluate true liver fibrosis. Lastly, there was a lack of structured longitudinal follow up in our study.

In conclusion, further characterization of GH in DM is needed. Resolution of hepatomegaly and normalized aminotransferases occurred while HbA1c remained high (but improved). GH appears to be associated with biochemical resolution without evidence of chronic liver injury. Simple steatosis is associated with GH in lean diabetic children. An intriguing question is why only a small group of children with poorly controlled type 1 DM develop GH. Increasing use of of intensive insulin therapy such as an insulin pump might improve or prevent GH in children. GH needs structured longitudinal follow‐up in the future.

## CONFLICT OF INTEREST STATEMENT

The authors declare no conflict of interest.
